# Nanoplatforms for Multimodal Imaging and Targeted Cancer Therapy: Recent Advances and Future Perspectives

**DOI:** 10.3390/bioengineering13020174

**Published:** 2026-02-02

**Authors:** Malairaj Sathuvan, Karthikeyan Narayanan, Kit-Leong Cheong, Ramar Thangam

**Affiliations:** 1 Centre for Advanced Studies in Botany, University of Madras, Guindy Campus, Chennai 600025, Tamil Nadu, India; 2 Center for Biotechnology and Interdisciplinary Studies, Rensselaer Polytechnic Institute, Troy, NY 12180, USA; narayk@rpi.edu; 3 Guangdong Provincial Key Laboratory of Aquatic Product Processing and Safety, College of Food Science and Technology, Guangdong Ocean University, Zhanjiang 524088, China

**Keywords:** metallic nanoplatforms, cancer theranostics, multimodal imaging, photothermal therapy, tumor targeting, nanoparticle biocompatibility

## Abstract

Recent advancements in nanotechnology have led to the development of multifunctional nanoplatforms that significantly enhance both cancer diagnosis and treatment. Gold-based nanoparticles, such as peptide-functionalized nanostructures and PEG-coated nanorods, offer improved tumor targeting, multimodal imaging (including photoacoustic and fluorescence), and effective photothermal therapy. Similarly, ultrafine iron oxide nanoprobes provide superior tumor imaging, while silver-based nanoparticles exhibit rapid systemic circulation, near-infrared fluorescence, and powerful photothermal properties. Titanium-based nanoplatforms enable a combination of therapies and advanced imaging methods. On the therapeutic side, polymeric nanoparticles (PNPs), silica-based platforms, PEG-based nanoparticles, and graphene oxide-based systems each offer unique advantages for targeted drug delivery and theranostics. PNPs, with tunable size, shape, and surface chemistry, enable controlled drug release and reduced side effects, while silica-based nanoplatforms improve tumor targeting and imaging. PEG-based nanoparticles enhance drug release and tumor penetration, and graphene oxide-based systems facilitate subcellular targeting and synergistic therapies. Collectively, these innovations are paving the way for more efficient, precise, and safer cancer therapies, leading to improved clinical outcomes.

## 1. Introduction

Cancer is characterized by the abnormal and uncontrolled proliferation of cells, often caused by genetic mutations or environmental factors like radiation and toxins. Poor lifestyle choices and aging also significantly increase cancer risk [1]. In recent decades, nanotechnology has garnered significant scientific attention due to its remarkable physical and functional properties. Nanoparticles (NPs) offer a promising and cost-effective approach in cancer therapy, owing to their unique physicochemical features—including small size, low toxicity, diverse chemical makeup, and a high surface-area-to-volume ratio [2]. To address these challenges, researchers are exploring advanced nanotechnology to develop innovative therapeutic approaches. Commonly utilized nanocarriers include dendrimers, nanoshells, polymeric and magnetic nanoparticles, liposomes, and nucleic acid-based nanostructures [3]. Metal-based nanoparticles are highly attractive in nanomedicine for their uniform size, prolonged activity, surface functionalization, and suitability for optical and thermal therapies. Their higher density also enhances cellular uptake, making them beneficial for cancer treatment [4]. Their strong bio-affinity and exceptional fluorescence make them ideal for visual detection, labeling, and applications in biological sensing, imaging, and therapy [5].

In the last ten years, advances in metallic nanoparticles—including gold, platinum, iron oxide, and bimetallic hybrids have transformed cancer imaging techniques such as CT, MRI, and photoacoustic imaging, while also facilitating treatment approaches like photothermal, photodynamic, and radiotherapy [6]. With millions of lives lost each year to cancer, the global impact of the disease highlights the critical need for more effective diagnostic and therapeutic approaches that offer high efficacy with reduced side effects [7]. The evolution from monofunctional agents to multifunctional nanoplatforms reflects a trajectory toward real-time imaging-guided therapy and synergistic treatment modalities [8]. Despite significant advances, challenges remain in achieving clinically translatable metallic nanoplatforms that combine high biocompatibility, targeted delivery, and multimodal imaging with effective therapeutic outcomes [9]. Controversies exist over the relative merits of different metallic compositions and hybrid structures, with some studies emphasizing gold-based platforms for their optical properties and others highlighting platinum or bimetallic alloys for enhanced catalytic and radio-sensitizing effects [10,11]. The consequences of these gaps include suboptimal therapeutic efficacy and safety profiles, hindering the advancement of personalized cancer theranostics [6].

The conceptual framework underpinning this review defines metallic nanoplatforms as nanoscale constructs composed of metals or metal alloys engineered for simultaneous cancer imaging and therapy [12]. This review employs a rigorous methodology encompassing a critical appraisal of recent literature on metallic and polymeric nanoplatforms in cancer theranostics, with inclusion criteria focusing on multimodal imaging and combined therapeutic applications. Analytical frameworks assess physicochemical characteristics, biological interactions, and clinical relevance. The findings are organized thematically to cover synthesis methods, imaging modalities, and therapeutic strategies considerations.

## 2. Metal Nanoparticle for Cancer Imaging and Therapy

Nanomaterials with diverse morphologies have been widely explored for cancer therapy and diagnostic imaging. These nanostructures can serve multiple functions, including drug delivery carriers, imaging contrast agents, photothermal and photoacoustic agents, as well as radiation dose enhancers, thereby enabling integrated therapeutic and diagnostic (theranostic) applications [13]. Metallic nanoparticles, including gold (Au), silver (Ag), platinum (Pt), zinc-based nanoparticles, and titanium dioxide (TiO_2_), have shown considerable potential in cancer diagnosis and therapy. Their unique magnetic, optical, thermal, and electrical properties enable diverse applications such as imaging, drug delivery, photothermal therapy, and radio-sensitization. Among these, gold nanoparticles and magnetic nanoparticles, particularly iron oxide (Fe_3_O_4_), have attracted significant attention due to their favorable biocompatibility, imaging capability, and therapeutic efficiency in anticancer applications [14]. Beyond Au, Fe, Ag, Ti, Cu, and Pt, several other metallic nanoplatforms have emerged as powerful cancer theranostic agents, notably those based on Mo, Ce, Pd, Gd, Mn, and Eu. Palladium-based nanomaterials, including Pd nanosheets, porous Pd nanoparticles, and Pd-based nanocomposites, exhibit strong near-infrared (NIR) absorption with exceptional photothermal conversion, superior photothermal stability, and high X-ray attenuation, enabling multimodal photoacoustic/CT/MRI imaging-guided photothermal therapy, radiotherapy enhancement, and chemotherapy [15]. These emerging metallic nanoplatforms substantially expand the theranostic toolbox beyond conventional metals, offering unique physicochemical properties tailored to specific imaging modalities and therapeutic mechanisms that address limitations such as tumor hypoxia, drug resistance, and imaging depth penetration in precision oncology.

### 2.1. Gold Nanomaterials for Cancer Imaging and Therapy

**Figure 1:**
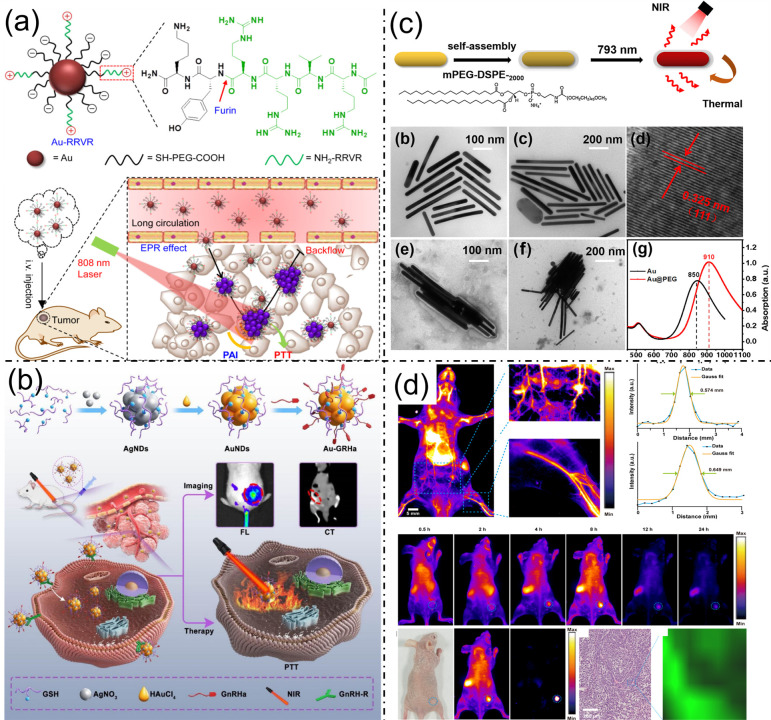
(**a**). Schematic of Au-RRVR decorated with furin-cleavable RVRR peptides and its in vivo behavior for tumor photoacoustic imaging and photothermal therapy. Reproduced with permission from ref. [16]. Copyright American Chemical Society 2021. (**b**) Diagrammatic representation of the Au-GRHa nanosystem designed for dual-mode imaging-guided, tumor-targeted photothermal therapy. Reproduced with permission from ref. [17]. Copyright American Chemical Society 2025. (**c**) Preparation and characterization process of Au@PEG nanorods, subfigures (**b**–**d**) show the different magnification TEM images of Au@PEG nanorods. (**e**,**f**) structures of Au@PEG nanorods. (**g**) UV–vis curve of Au@PEG nanorods. Reproduced with permission from ref. [18]. Copyright American Chemical Society 2021. (**d**) NIR-IIb imaging of mouse abdominal vessels and femoral artery with intensity profiles, tumor uptake of JNPs@DTTC over time, in vivo/ex vivo tumor fluorescence, and H&E-stained and Raman tumor images. Reproduced with permission from ref. [19]. Copyright American Chemical Society 2025.

Advancements in nanotechnology have led to the creation of multifunctional gold-based nanomaterials that improve tumor-targeted imaging and treatment by leveraging the distinctive characteristics of the tumor microenvironment. One notable approach involves gold nanoparticles (AuNPs) functionalized with furin-cleavable peptides, designed to respond synergistically to elevated furin enzyme levels and acidic pH characteristic of tumors. This dual-triggered aggregation markedly improves photoacoustic (PA) imaging sensitivity through enhanced near-infrared (NIR) absorption, while simultaneously activating photothermal therapy (PTT) for effective tumor ablation, providing a robust platform for precise cancer diagnosis and treatment (Figure 1a) [16]. Complementing this, the ultrasmall Au-GRHa nanosystem integrates fluorescence (FL) and computed tomography (CT) dual-mode imaging for early and accurate ovarian cancer detection. Functionalized with gonadotropin-releasing hormone peptides, this nanosystem exhibits enhanced tumor targeting via GnRH receptor binding, facilitating improved nanoparticle accumulation and enabling real-time intraoperative navigation and PTT with reduced systemic toxicity (Figure 1b) [17]. Similarly, gold nanorods (Au NRs) modified with polyethylene glycol (Au@PEG NRs) demonstrate excellent biocompatibility and photothermal conversion efficiency under NIR-I irradiation. Polyethylene glycol (PEG) coating reduces the toxicity associated with conventional stabilizers, enabling the safe and effective destruction of cervical cancer cells while offering strong potential as a theranostic platform for targeted imaging and treatment (Figure 1c) [18]. Further expanding the multifunctionality of gold-based nanomaterials, Janus Au-PbS nanoparticles have been synthesized via epitaxial growth to integrate tunable second near-infrared (NIR-II) photoluminescence with surface-enhanced Raman scattering (SERS). This dual-modal imaging capability offers complementary tumor visualization, enabling precise intraoperative tumor resection and highlighting the promise of Janus nanoparticles for advanced bioimaging-guided cancer surgery (Figure 1d) [19]. Collectively, these innovations underscore the potential of engineered gold nanostructures as versatile platforms for enhancing the accuracy and efficacy of cancer diagnosis and treatment.

### 2.2. Silver-Based Nanoparticles in Cancer Theranostics

**Figure 2:**
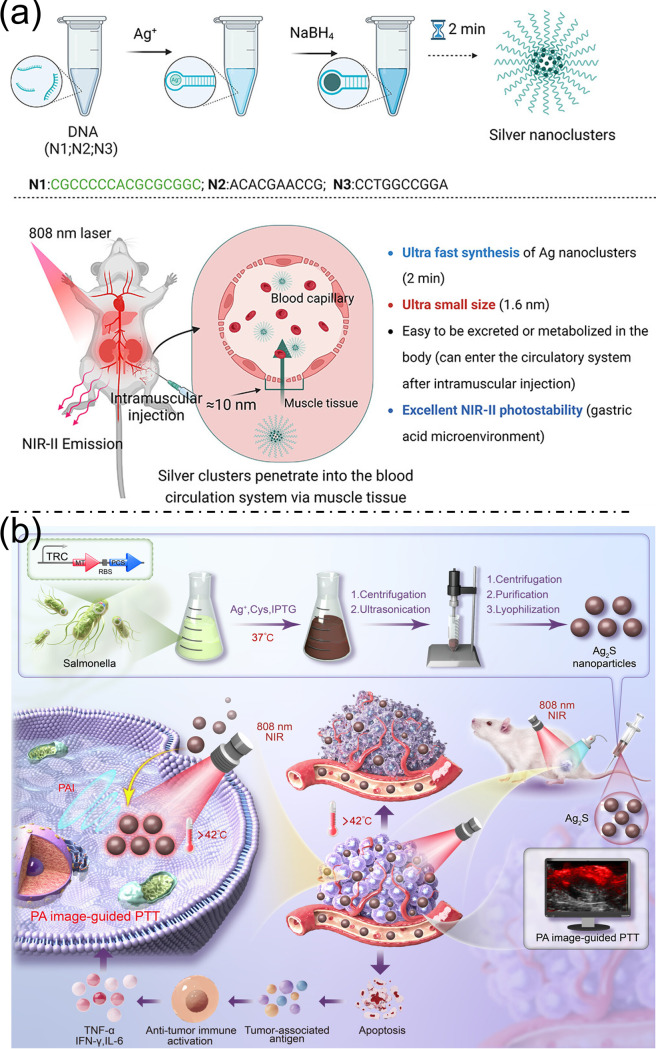
(**a**). Synthesis of DNA-templated silver nanoclusters (DNA)_2_(Ag^0^)_21_(Ag^+^)_9_ and a schematic showing in situ visualization of their metabolism in the bloodstream following intramuscular injection. Reproduced with permission from ref. [20]. Copyright American Chemical Society 2025. (**b**) Schematic of Ag_2_S nanoparticle biosynthesis in YB1 for imaging-guided photothermal therapy and antitumor immunity, showing nanoparticle formation from AgNO_3_ and L-cysteine and immune activation via tumor antigen release. Reproduced with permission from ref. [21]. Copyright American Chemical Society 2025.

Near-infrared-II (NIR-II) imaging offers high-resolution visualization of deep tissues with reduced autofluorescence and scattering, yet traditional nanoprobe synthesis is often slow and inefficient. To address this, DNA-templated silver nanoclusters (Ag NCs) approximately 1.6 nm in size have been efficiently synthesized via a simple two-minute, single-step procedure, enabling easy entry into muscle capillaries and efficient systemic circulation. After intramuscular injection in mice, these Ag NCs exhibited strong NIR-II fluorescence that gradually decreased over time, with significant accumulation observed in the liver and kidneys, likely due to in vivo aggregation and interaction with biological molecules. Compared to larger Ag_2_S quantum dots, which showed stable fluorescence but limited organ accumulation, the ultrasmall Ag NCs demonstrated superior penetration and clearance, suggesting enhanced biocompatibility and reduced long-term retention. This rapid metabolism reduces potential toxicity concerns, positioning these Ag NCs as promising, safe probes for biological imaging and advancing the clinical translation of nanoparticle-based nanomedicine (Figure 2a) [20].

Photothermal therapy (PTT) is widely recognized as an efficient cancer therapy method; however, traditional synthesis of PTT agents often involves complex procedures and environmentally harmful solvents. Addressing these challenges, this study introduces an innovative and eco-friendly biosynthetic approach utilizing genetically modified bacteria for the controlled production of Ag_2_S nanoparticles (30–80 nm) under mild conditions. These biosynthesized nanoparticles serve as promising theranostic agents for both photoacoustic imaging (PAI) and PTT because of their excellent biocompatibility, stability, and minimal toxicity. Notably, Ag_2_S nanoparticles exhibited excellent photoacoustic signals in the 680–970 nm range, with a peak excitation at 700 nm, aligning well with the optical window for deep tissue imaging. Tumor vasculature characteristics, such as irregular structures and enlarged pores, allow optimal accumulation of nanoparticles sized between 50 and 100 nm via the facilitated permeability and retention effect, and the 54 nm Ag_2_S nanoparticles efficiently localized at tumor sites. In vivo PAI of 4T1 tumor-bearing mice demonstrated a steady increase in photoacoustic signal intensity over time, peaking at 8 h post-intravenous-injection, confirming effective tumor targeting and retention. This imaging window coincides with the optimal timing for photothermal therapy. Upon laser irradiation (808 nm, 10 min), tumors treated with Ag_2_S nanoparticles showed a rapid temperature increase (23.5 °C), significantly higher than controls (4.7 °C), highlighting their superior photothermal conversion efficiency and promise for efficient tumor ablation. Collectively, these findings highlight that biosynthesized Ag_2_S nanoparticles combine excellent photoacoustic imaging capabilities with potent photothermal therapeutic effects, offering a sustainable and clinically translatable platform for cancer diagnosis and treatment (Figure 2b) [21].

### 2.3. Multimodal Imaging Using Iron Oxide Nanoprobes

**Figure 3:**
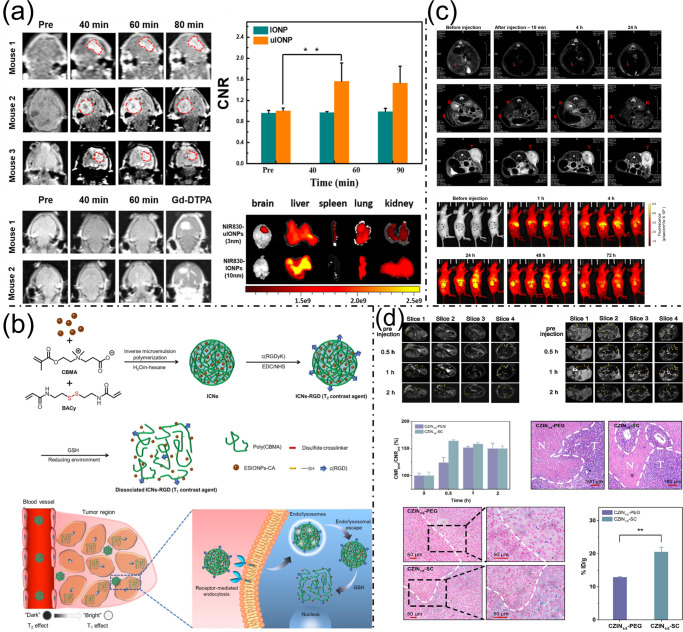
(**a**). T1-weighted MRI comparing sub-5 nm uIONPs and larger IONPs in brain tumors, showing time-dependent contrast enhancement, with NIR fluorescence tracking nanoparticle distribution in the brain and organs. Reproduced with permission from ref. [22] Copyright American Chemical Society 2022. (**b**) Preparation of ESIONP-poly(CBMA) nanogels with c(RGD) that switch MRI contrast from T2 to T1 in response to GSH, enabling precise tumor diagnosis using ICNs-RGD. Reproduced with permission from ref. [23]. Copyright American Chemical Society 2020. (**c**) MRI and NIR imaging of tumor-bearing mice after DiI/DiR/MNP injection, showing T2-weighted MRI of major organs and tumor before/after injection, and time-dependent tumor fluorescence signals. Reproduced with permission from [24]. Copyright American Chemical Society 2020. (**d**) Detection of liver metastases by T2-weighted MRI at 0–2 h after CZIN1/5-PEG or CZIN1/5-SC injection, with CNR analysis over time, and H&E/Prussian blue staining showing iron accumulation in metastases. Reproduced with permission from ref. [25]. Copyright American Chemical Society.

Ultrafine sub-5 nm iron oxide nanoparticles (uIONPs) coated with oligosaccharides have emerged as promising agents for enhanced molecular imaging of brain tumors. Their small size enables efficient crossing of the blood-brain and blood-tumor barriers in glioblastoma models, exploiting the tumor’s leaky vasculature for improved tumor accumulation. This leads to strong, time-dependent T1-weighted MRI contrast enhancement, peaking around 40 to 60 min after injection. Compared to larger nanoparticles, uIONPs demonstrate superior tumor penetration and retention, resulting in clearer and more precise tumor visualization while sparing normal brain tissue. Their renal clearance and minimal off-target accumulation underscore their safety and efficacy, positioning them as valuable tools for noninvasive, high-resolution imaging critical for accurate diagnosis and treatment planning (Figure 3a) [22]. To further refine MRI specificity, activatable agents responsive to tumor microenvironments have been developed. A notable example is a system comprising tremendously small iron oxide nanoparticles (ESIONPs) coated with citric acid and encapsulated in disulfide-cross-linked poly (carboxybetaine methacrylate) nanogels, functionalized with the tumor-targeting cyclic peptide c(RGD), forming ICNs-RGD. This design harnesses elevated glutathione (GSH) levels within tumors to induce disassembly of clustered ESIONPs into dispersed particles, switching their MRI contrast from T2 to T1 mode selectively within tumor cells and tissues. In vivo experiments confirmed that ICNs-RGD enhances T1-weighted contrast specifically at tumor sites following intravenous administration, benefiting from both GSH-triggered activation and tumor-targeting capabilities. This combination presents a promising approach for precise and efficient tumor imaging in clinical contexts (Figure 3b) [23].

Integrating MRI with fluorescence imaging (FI) offers a versatile platform for comprehensive disease detection and image-guided therapy. A recent advancement involves a modular lipid-based assembly strategy to generate a diverse library of MRI/FI nanoprobes by combining magnetic nanoparticles (MNPs), fluorescent dialkylcarbocyanine dyes, and targeting ligands through three orthogonal processes: encapsulation within phospholipid and phospholipid-PEG layers, physical adsorption of fluorescent dyes, and surface ligand conjugation. This independent tuning allows control over nanoprobe size, fluorescence emission wavelengths, and targeting properties. The resulting DiI/DiR/MNP nanoprobes exhibit remarkable stability, biocompatibility, and strong contrast signals in whole-body MRI, near-infrared fluorescence imaging, and microscopy. Their PEG coating enhances biological stability and facilitates ligand conjugation, increasing cellular uptake. Sized between 20 and 50 nm, these probes effectively penetrate tumor vasculature through the enhanced permeability and retention effect, rendering them ideal for tumor diagnosis, intraoperative imaging, and biopsy guidance. This lipid-based nanofabrication method thus provides a powerful, customizable platform for multimodal imaging tailored to diverse biomedical applications (Figure 3c) [24]. Advancements in MRI contrast enhancement also focus on optimizing T2 agents. Cubic zinc-doped iron oxide nanoparticles (CZINs) synthesized via thermal decomposition, with varying zinc-to-iron ratios, revealed that CZINs doped at a 1:5 ratio possessed the highest magnetic saturation (63.3 emu/g) and superior T2 relaxivity. Surface modifications further influenced their in vivo distribution and targeting efficiency. PEG-modified CZINs demonstrated reduced protein adsorption, prolonged circulation time, and lower liver uptake, making them ideal for accurate imaging of subcutaneous tumors. Conversely, sodium citrate (SC)-modified CZINs exhibited rapid clearance and preferential liver accumulation, enhancing the detection accuracy of liver metastases. These findings emphasize how optimizing both crystal structure and surface chemistry can tailor MRI contrast agents for specific tumor types, advancing the development of clinically translatable T2-weighted contrast probes (Figure 3d) [25].

### 2.4. Titanium Nanoplatforms for Cancer Imaging and Therapy

**Figure 4:**
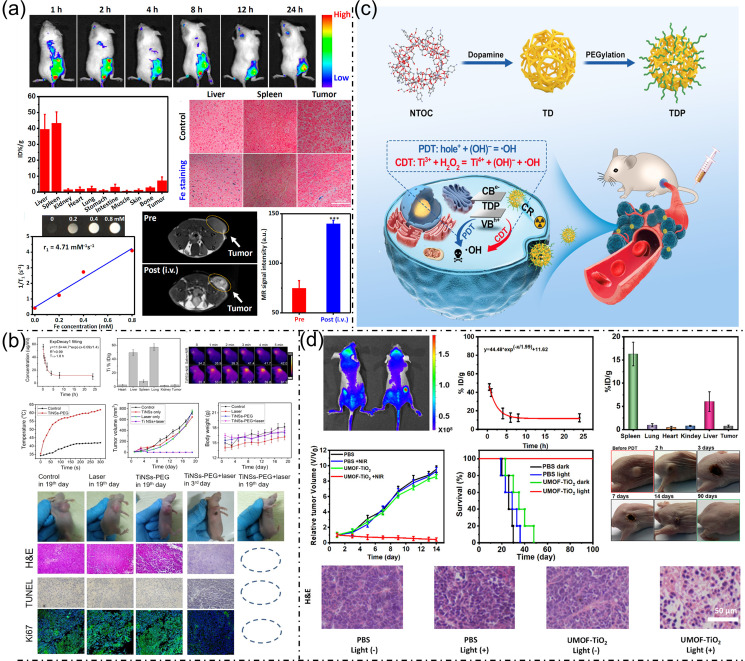
(**a**). In vivo multimodal imaging of 4T1 tumor-bearing mice after Fe-TiO_2_ ND injection, showing fluorescence tracking, Ti biodistribution at 24 h, Prussian blue–confirmed organ accumulation, and concentration-dependent MRI tumor contrast. Reproduced with permission from ref. [26]. Copyright American Chemical Society 2020. (**b**) In vivo photothermal therapy with TiNSs-PEG, showing circulation and biodistribution, infrared-confirmed tumor heating under laser irradiation, tumor growth and body weight changes, and histological evaluation (H&E, TUNEL, Ki-67). Reproduced with permission from ref. [27]. Copyright American Chemical Society 2019. (**c**) Schematic showing TDP as a multifunctional nanoplatform for CR-triggered synergistic photo- and chemodynamic therapy. Reproduced with permission from ref. [28]. (**d**) Fluorescence imaging of UMOF-TiO_2_ in tumor-bearing mice with/without intratumoral injection, blood metabolism via Zr tracking, biodistribution after intravenous injection, tumor volume and survival over 100 days, acute PDT-induced tumor regression, and H&E histology. Reproduced with permission from ref. [29]. Copyright American Chemical Society 2020.

Recent progress in nanomedicine has markedly improved the effectiveness of cancer treatments by integrating multifunctional nanoplatforms with imaging and treatment capabilities. Sonodynamic therapy (SDT), a noninvasive technique leveraging ultrasound (US) to activate sonosensitizers and generate reactive oxygen species (ROS) for tumor ablation, has seen notable improvements through the development of ultrasmall iron-doped titanium dioxide nanodots (Fe-TiO_2_ NDs). Produced through thermal decomposition, these nanodots exhibit enhanced ROS production under US stimulation, attributed to iron doping, which also imparts Fenton-like catalytic activity for chemodynamic therapy (CDT) by converting tumor-derived H_2_O_2_ into ROS. PEGylated Fe-TiO_2_-PEG nanodots demonstrate excellent stability, biocompatibility, and tumor retention, verified by magnetic resonance (MR) and fluorescence imaging, with a remarkable 1.87-fold increase in tumor signal intensity in T1-weighted MR imaging. This dual CDT and SDT approach results in superior therapeutic outcomes relative to commercial TiO_2_ nanoparticles. At the same time, efficient clearance within one month minimizes long-term toxicity, underscoring their promise as safe, multifunctional theranostic agents (Figure 4a) [26].

Two-dimensional titanium nanosheets (TiNSs) also provide a multifunctional platform with impressive imaging features for cancer theranostics. Exfoliated from bulk titanium, TiNSs exhibit strong near-infrared absorption and superior photothermal conversion efficiency, but their metallic nature further enables robust contrast for photoacoustic (PA) and computed tomography (CT) imaging. PEGylated TiNSs demonstrated effective tumor accumulation via the enhanced permeability and retention (EPR) effect, with PA imaging revealing a gradual increase in signal intensity at the tumor site, peaking at 24 h post-injection. This temporal PA signal tracking reflects real-time tumor targeting and nanomaterial metabolism. CT imaging corroborated these findings, showing enhanced X-ray attenuation at tumor sites 24 h after administration, providing clear tumor visualization. The dual-modal imaging approach offered by TiNSs enhances tumor delineation and facilitates image-guided photothermal therapy (PTT), promoting precision treatment with real-time feedback. These imaging properties, combined with biocompatibility and efficient metabolism, make TiNSs a valuable tool in transition-metal-based cancer theranostics (Figure 4b) [27].

The effectiveness of photodynamic therapy (PDT) is frequently constrained by the limited penetration depth of external light into tissues. However, the use of Cerenkov radiation (CR) from radioactive isotopes provides an internal excitation source, enhancing imaging-guided therapy. In this context, NH_2_-Ti_32_O_16_ nanocluster (NTOC)-derived ultrasmall nanophotosensitizers (TDPs) were engineered with dopamine (DA) ligands to improve solubility and tumor targeting via DA receptor affinity. These TDPs exhibit efficient CR-induced type I PDT and chemodynamic therapy. However, importantly, their ultrasmall size and surface modification also favor favorable pharmacokinetics and tumor accumulation, critical for effective imaging. Although direct imaging modalities such as MR or fluorescence were not the primary focus, the high tumor targeting and extended blood circulation observed imply strong potential for coupling with CR-based imaging or nuclear imaging techniques. The combination of CR excitation and targeted delivery positions TDPs as promising agents in nuclear medicine theranostics, enabling simultaneous imaging and therapy with minimal systemic toxicity (Figure 4c) [28].

Addressing limitations in conventional PDT, such as poor ROS generation and tissue penetration, a novel multimodal nanoplatform (UMOF-TiO_2_) was developed by integrating ultrasmall TiO_2_ nanoparticles onto a heterodimer of upconversion nanoparticles (UCNPs) and porphyrin-based metal–organic frameworks (MOFs). This design leverages UCNPs to convert NIR light into UV and visible light, activating both Type I and Type II PDT mechanisms while also enabling intrinsic fluorescence imaging via the porphyrin molecules. In vivo fluorescence imaging demonstrated clear tumor localization with significant signal contrast, confirming effective tumor accumulation of UMOF-TiO_2_. Pharmacokinetic studies revealed a blood half-life of 1.99 h and dominant biodistribution in the liver and spleen. The ability to image tumors through fluorescence provides a powerful tool for noninvasive monitoring of therapeutic delivery and efficacy. Combined with the platform’s potent photodynamic therapy performance and minimal systemic toxicity, UMOF-TiO_2_ stands out as a robust agent for integrated cancer imaging and treatment with clinical translation potential (Figure 4d) [29].

### 2.5. Copper Sulfide Nanoplatforms for Cancer Imaging and Therapy

Recent advances in multifunctional nanotheranostics have demonstrated promising strategies for integrating cancer diagnosis and therapy within a single platform. Mansoorianfar et al. (2023) [30] reported the development of bandgap-modified Fe_3_O_4_@Ag@CuS nanoparticles functionalized with the AS1411 DNA aptamer (aptaNPs), combining photothermal therapy (PTT) with dual magnetic resonance (MR) and infrared (IR) imaging. These aptaNPs overcome the limitations of conventional treatments by integrating targeted therapy and diagnostic imaging. Notably, the nanoparticles exhibit strong localized surface plasmon resonance (LSPR), achieving a high light-to-heat conversion efficiency of 34% and a mass extinction coefficient of 1.97 L·cm^−1^·g^−1^. Both in vitro and in vivo studies confirmed enhanced uptake by 4T1 cancer cells via aptamer-mediated targeting, with MR imaging validating effective tumor accumulation. Histological analyses revealed good biosafety, with gradual metabolism and no observable organ toxicity, highlighting the potential of aptaNPs for precise imaging-guided photothermal ablation of deep-seated or advanced tumors.

**Figure 5:**
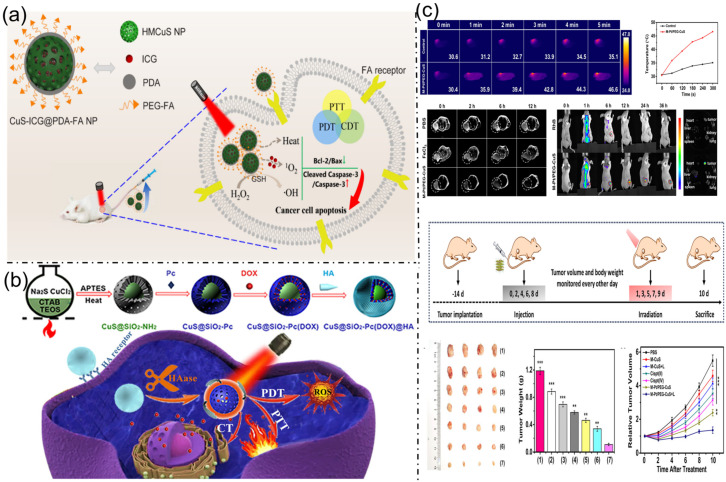
(**a**). A schematic representation of CuS-ICG@PDA-FA nanoparticles designed for efficient targeted cancer therapy with near-infrared laser-activated photothermal, photodynamic, and chemodynamic effects. Reproduced with permission from ref. [31]. Copyright 2025 American Chemical Society. (**b**) Imaging of SK-OV-3 tumors 12 h after PBS or M-Pt/PEG-CuS injection under 1064 nm laser, showing temperature changes, T2-weighted MRI, fluorescence, schematic of laser treatment, and excised tumor images with weights and volumes. Reproduced with permission from ref. [32]. Copyright 2023 American Chemical Society. (**c**) Illustration of the Synthetic Pathway and Anticancer Mechanism of CuS@mSiO_2_-Pc(DOX)@HA Nanoparticles. Reproduced with permission from ref. [33]. Copyright 2022 American Chemical Society.

Multifunctional copper sulfide–based nanoplatforms have gained increasing attention for synergistic cancer therapy due to their intrinsic photothermal properties and catalytic activity for chemodynamic therapy. Hollow mesoporous CuS nanoparticles provide high drug-loading capacity and efficient photothermal conversion, while simultaneously enabling Fenton-like reactions to generate reactive oxygen species in tumor microenvironments. The incorporation of photosensitizers such as indocyanine green further enhances photodynamic performance under near-infrared irradiation. Surface modification with biocompatible polymers, including polydopamine, improves nanoparticle stability and facilitates subsequent functionalization. Targeting ligands such as folic acid enable selective tumor cell uptake through receptor-mediated endocytosis. Together, these integrated strategies enable effective multimodal photothermal, photodynamic, and chemodynamic therapies with improved antitumor efficacy and biosafety (Figure 5a) [31]. Similarly, Qiang et al. (2022) [32] developed a multifunctional M-Pt/PEG-CuS nanosystem for ovarian cancer therapy, integrating chemotherapy and PTT within a single platform. Constructed from ultrasmall CuS-modified Fe(III)-MOFs and a cisplatin prodrug, this system enables MRI-guided, tumor-specific treatment. Following intravenous administration, M-Pt/PEG-CuS selectively accumulated at tumor sites and, under 1064 nm laser irradiation, achieved synergistic tumor inhibition while minimizing toxicity to major organs. These findings underscore the platform’s efficiency and safety, demonstrating its potential for clinical translation in ovarian cancer treatment (Figure 5b). Lv et al. (2022) [33] presented a CuS@mSiO_2_-Pc(DOX)@HA nanoplatform designed for targeted therapy of triple-negative breast cancer (TNBC) through combined photothermal therapy (PTT), photodynamic therapy (PDT), and chemotherapy (CT). The nanoparticles consist of CuS cores coated with Zn-phthalocyanine-functionalized mesoporous silica for phototherapy, loaded with doxorubicin (DOX) for chemotherapy, and capped with hyaluronic acid (HA) for tumor targeting. This design affords high drug-loading capacity, controlled HAase-triggered DOX release, efficient ROS generation, and photothermal conversion under near-infrared irradiation. In vitro studies revealed strong cytotoxicity against 4T1 cells, confirming the platform’s synergistic therapeutic efficacy. This targeted, multimodal system holds significant promise for minimally invasive TNBC treatment, with future in vivo studies aimed at clinical translation (Figure 5c).

### 2.6. Platinum Nanoplatforms for Cancer Imaging and Therapy

**Figure 6:**
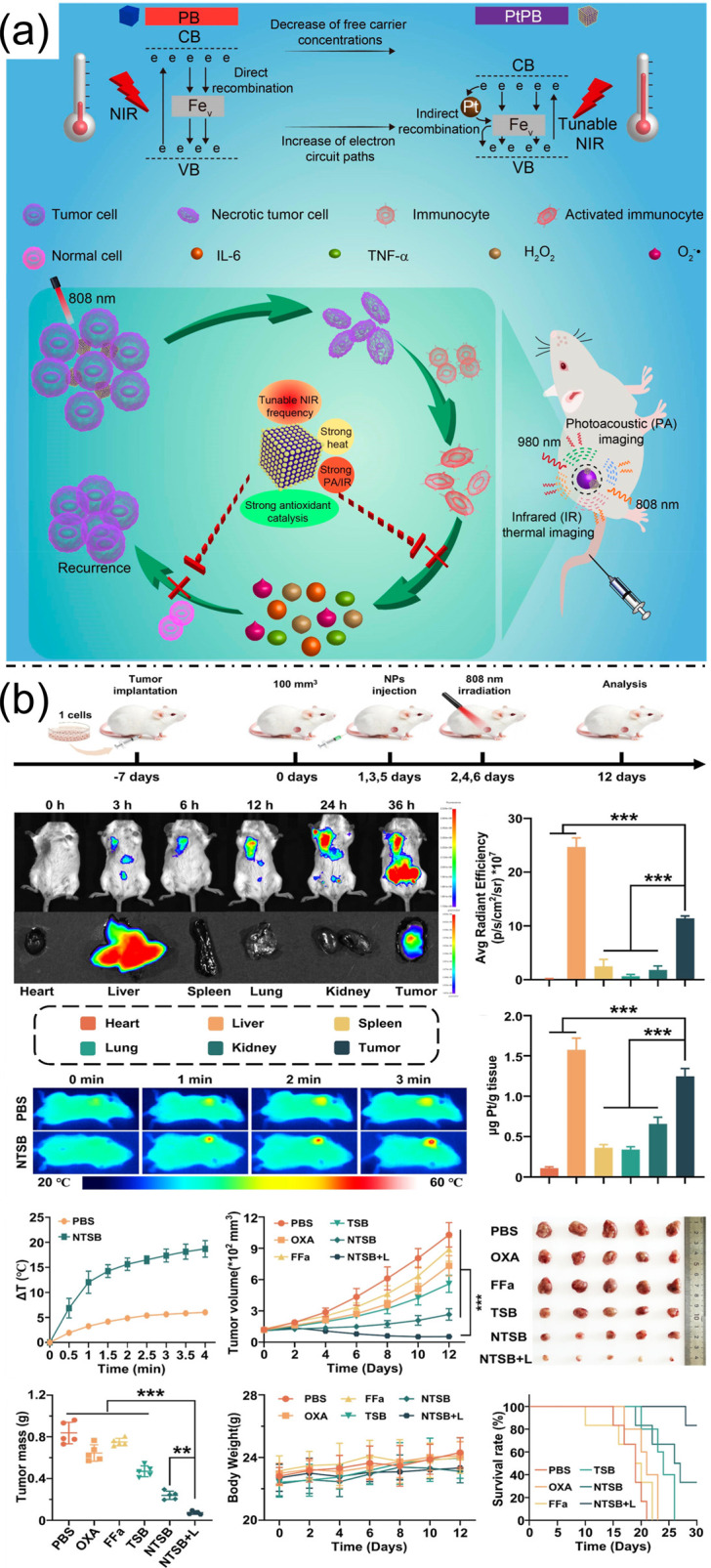
(**a**). Pt-doped PB nanocubes for enhanced photothermal properties, multiwavelength PA/IR imaging, and imaging-guided photothermal therapy with inflammation relief. Reproduced with permission from ref. [35]. Copyright 2021 American Chemical Society. (**b**) In vivo biodistribution and antitumor efficacy of NTSB in 4T1 tumor-bearing mice, showing tumor/organ accumulation (fluorescence and ICP–MS), NIR-induced temperature elevation, tumor growth inhibition (tumor curves, images, weights), stable body weight, and 30-day survival, demonstrating therapeutic efficacy and biosafety. Reproduced with permission from ref. [36]. Copyright 2025 American Chemical Society.

Recent advances in nanomedicine have highlighted multifunctional nanozyme-based platforms for synergistic tumor therapy and imaging. Ti_3_C_2_Tx nanosheets decorated with platinum nanoparticles (Ti_3_C_2_Tx-Pt-PEG) exhibit peroxidase-like activity, converting H_2_O_2_ into cytotoxic hydroxyl radicals, which is enhanced under NIR-II-induced hyperthermia, achieving a photothermal conversion efficiency of 31.78% and enabling effective tumor ablation with photoacoustic-guided therapy [34]. Similarly, platinum-doped Prussian blue (PtPB) nanozymes with tunable localized surface plasmon resonance demonstrate enhanced photothermal conversion efficiency (58.2%) and antioxidative catalytic activity, alleviating PTT-induced inflammation while achieving strong tumor inhibition (Figure 6a) [35]. Platinum-based anticancer agents typically act by forming adducts with nuclear DNA, inhibiting transcription, and inducing apoptosis; however, tumor cells often develop resistance. Targeting mitochondrial DNA (mtDNA), which lacks nucleotide excision repair, provides an alternative strategy. A novel platinum-based terminal-sensitive projectile (TSB) was developed, consisting of a tetravalent platinum prodrug as the primary warhead, guided by triphenylphosphine (TPP) and a secondary warhead, fenofibric acid (FFa). Encapsulation of TSB within IR780-conjugated DSPE-PEG2K (NTSB) facilitates precise mitochondrial targeting, releasing oxaliplatin and FFa to cross-link mtDNA and disrupt the electron transport chain, respectively. Near-infrared irradiation of the IR780 component generates heat and reactive oxygen species, depleting glutathione and enhancing platinum–mtDNA interactions. This integrated strategy significantly improves tumor cell sensitivity to platinum chemotherapy in both in vitro and in vivo models. (Figure 6b) [36].Collectively, these platinum-based nanoplatforms integrate high-efficiency photothermal therapy, catalytic tumor ablation, and noninvasive imaging, offering precise and safe strategies for cancer treatment.

### 2.7. Manganese-Based Nanoplatforms for Cancer Imaging and Therapy

**Figure 7:**
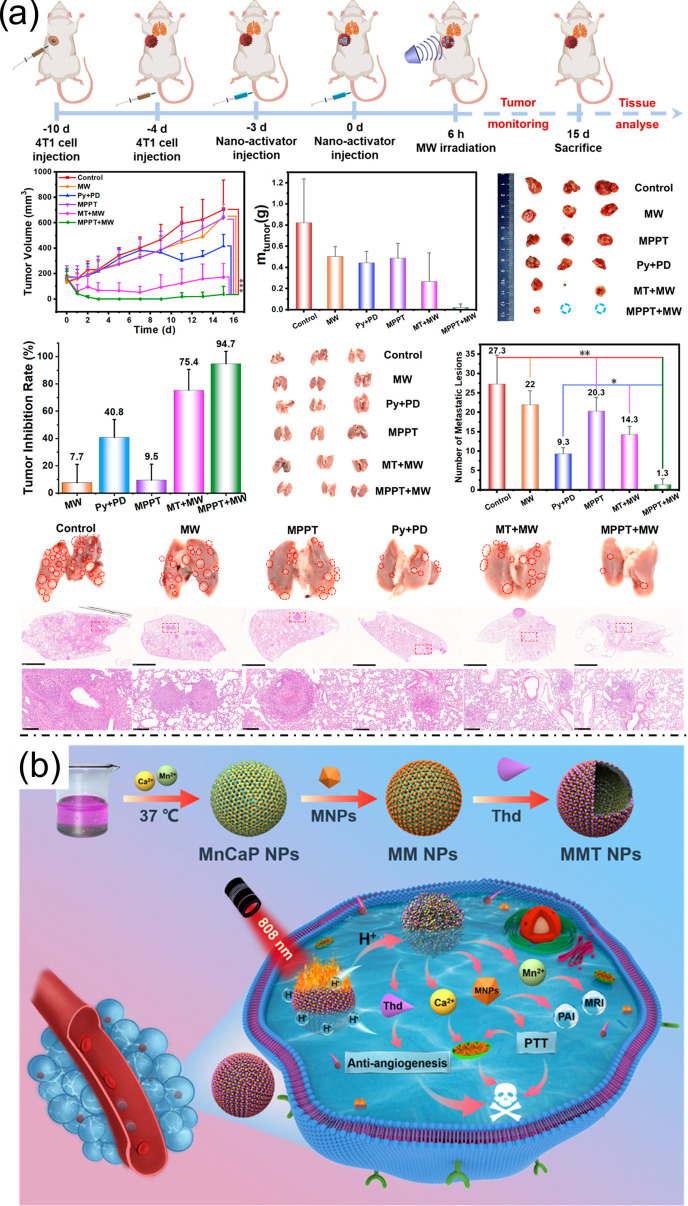
(**a**) In vivo evaluation of MPPT NAs for metastatic tumor treatment, showing primary tumor growth curves, tumor weights, representative images, inhibition rates, and lung metastasis assessment by imaging, nodule counting, and H&E staining, demonstrating suppression of both primary and metastatic tumors. Reproduced with permission from ref. [38]. Copyright 2023 American Chemical Society. (**b**) Synthesis of Hollow MMT Nanoparticles and pH-/NIR-Responsive Drug Release for Combined Photothermal and Anti-Angiogenic Tumor Therapy. Reproduced with permission from ref. [39]. Copyright 2022 American Chemical Society.

Recent advances in multifunctional nanoplatforms have enabled synergistic strategies to enhance anticancer efficacy by combining multiple therapeutic modalities. Chemodynamic therapy (CDT), which generates hydroxyl radicals (•OH) through metal-ion-mediated hydrogen peroxide decomposition, has shown promise. However, its effectiveness is often limited by high intracellular glutathione (GSH) levels. Dual GSH-depleting nanosystems, such as MnO_2_@PDA@PEG@DOX, have been developed to address this limitation, simultaneously enabling CDT, chemotherapy, and sonodynamic therapy (SDT) under ultrasound irradiation, thereby significantly improving tumor suppression [37]. To tackle tumor recurrence and metastasis, especially in aggressive cancers like triple-negative breast cancer (TNBC), multifunctional metal–organic framework (MOF)-based nano-activators have been engineered to integrate chemotherapy, thermodynamic therapy, and immunotherapy. MPPT nano-activators co-deliver chemotherapeutic agents (e.g., Pyrotinib) and immune checkpoint inhibitors (PD-1/PD-L1), while targeting tumors via triphenylphosphine (TPP) and the enhanced permeability and retention (EPR) effect. Under microwave irradiation, these nanosystems not only ablate primary tumors but also modulate the immunosuppressive tumor microenvironment through Mn^2+^-mediated cGAS-STING activation, enhancing immune infiltration and synergizing with pathway inhibition (PI3K/AKT/mTOR and MAPK/ERK) to suppress metastases (Figure 7a) [38]. Similarly, photothermal therapy (PTT) combined with anti-angiogenic therapy has been explored using biodegradable hollow manganese-doped calcium phosphate nanoparticles (MnCaP)-MNP-Thd (MMT). These nanosystems integrate melanin nanoparticles for PTT and thalidomide for anti-angiogenesis, exhibiting dual-responsive release under acidic conditions and near-infrared irradiation. The combined effects of mitochondrial Ca^2+^ overload, angiogenesis inhibition, and photothermal ablation lead to enhanced tumor suppression, with the added benefit of multimodal imaging via Mn^2+^-enabled MRI and MNP-mediated photoacoustic imaging (Figure 7b) [39].

Jointly, these developments underscore the critical importance of imaging in both the design and implementation of titanium-based nanotherapeutics. By incorporating magnetic resonance, photoacoustic, computed tomography, fluorescence, and nuclear imaging functionalities, these nanoplatforms enable precise tumor localization, treatment monitoring, and improved therapeutic outcomes (Table 1). The convergence of enhanced imaging contrast with therapeutic modalities not only facilitates real-time guidance and assessment but also fosters the development of personalized cancer theranostics, improving safety and efficacy in clinical oncology.

**Table 1:** Advanced metallic nanoplatforms for cancer theragnostics.

Nano Platform Design	Imaging Modalities Integration	Targeting Efficiency	Therapeutic Efficacy	Ref.
Pt-decorated metal–organic framework, nanoscale size, multimodal surface functionalization (NMOF545@Pt)	CT, MRI, and Photoacoustic imaging combined	Passive tumor accumulation demonstrated	Enhanced photothermal and radiotherapy synergy	[40]
Self-assembled 1D silver-coated gold nanochains, adaptive plasmonic structure (Au@Ag NPs)	Photoacoustic imaging with NIR absorption tuning	Tumor microenvironment-activated targeting	High photothermal conversion efficiency for PTT	[41]
Platinum-based nanoplatforms with electrocatalytic activity (Pt)	Multimodal imaging, including photothermal and radiotherapy	Image monitoring and drug delivery integration	Chemotherapy, electrodynamic, photothermal, immunotherapy	[10]
Iron-based hollow nanoplatforms (Fe-HNPs)	T1/T2 MRI and dual-modal imaging	Tumor microenvironment-responsive drug release	MRI-guided therapies with multifunctionality	[42]
Furin/acidic pH–responsive AuNPs (Au-RRVR)	Photoacoustic imaging	Enzyme- and pH-triggered tumor-specific aggregation	Aggregation-enhanced photothermal therapy	[16]
Ultrasmall Au-GRHa nanosystem functionalized with GnRHa	Fluorescence (FL) and CT imaging	GnRH-R–mediated active targeting to ovarian cancer cells	Photothermal therapy with reduced side effects vs. chemo/radiotherapy	[17]
Ultrasmall iron-doped TiO_2_ nanodots (Fe-TiO_2_-PEG)	MR and fluorescence imaging	Efficient tumor retention after intravenous injection	Combined sonodynamic and chemodynamic therapy via enhanced ROS generation	[26]
Two-dimensional titanium nanosheets (Ti NSs)	CT and photoacoustic (PA) imaging	Passive tumor accumulation with good biocompatibility	High-efficiency photothermal therapy (61.5% conversion efficiency)	[27]
Biosynthesized Ag_2_S nanoparticles	Photoacoustic imaging (PAI)	Efficient tumor accumulation with high biocompatibility	Photothermal therapy with 38.5% conversion efficiency and effective antitumor activity	[21]

## 3. Advances in Polymeric Nanoparticles for Cancer Therapy

Polymeric nanoparticles (PNPs) are nanoscale materials composed of natural or synthetic polymers, offering remarkable versatility in drug delivery applications. Their ability to be tailored in terms of size, shape, and surface chemistry differentiates them from other nanoparticle systems, making them ideal for encapsulating both hydrophilic and hydrophobic drugs with high efficiency [43]. Due to their unique characteristics, PNPs have become a preferred choice for targeted and theranostic drug delivery systems. Key benefits include biodegradability, biocompatibility, and the ability to control drug release, which makes them particularly suitable for cancer therapy [44]. These nanoparticles are capable of improving drug targeting and reducing side effects by encapsulating or conjugating therapeutic agents, thereby enhancing treatment outcomes [45]. In addition, PNPs offer advantages like non-toxicity, prolonged circulation, and the ability to carry diverse therapeutic agents, further contributing to their potential for improving the efficacy and safety of cancer treatments The tunable nature of PNPs also allows for precise control over drug release at specific locations in the body, making them especially useful for delivering water-soluble drugs or overcoming challenges such as short drug half-life (Table 2) [46].

### 3.1. Silica-Based Nanoplatforms in Cancer Theranostics and Targeted Therapy

**Figure 8:**
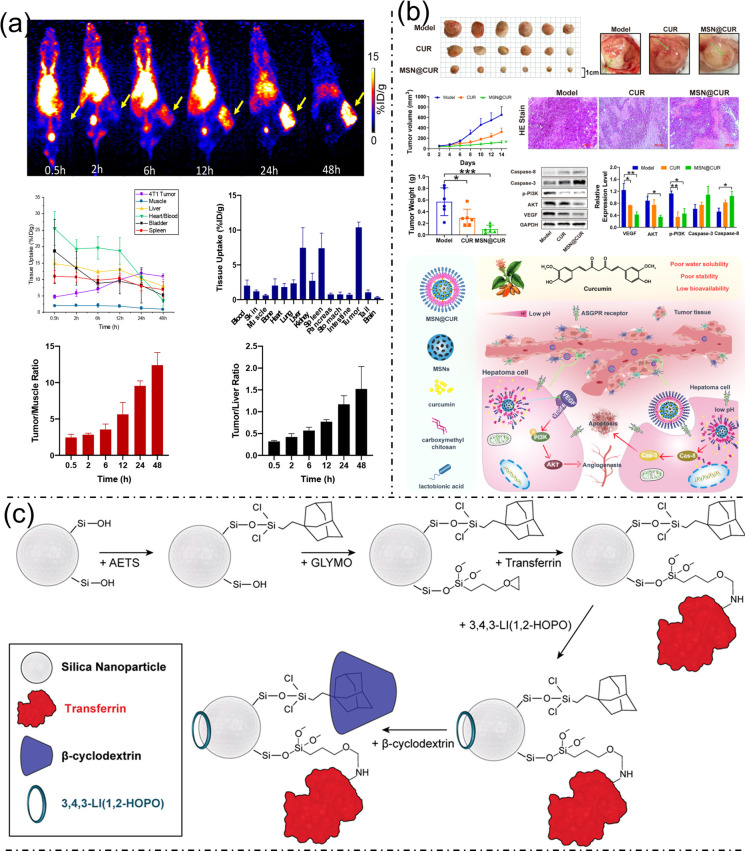
(**a**) In vivo PET imaging of 4T1 tumor-bearing mice after ^86Y-DOTA-UPSN administration, showing tumor localization, organ time–activity profiles, ex vivo biodistribution at 48 h post-injection, and quantitative tumor-to-muscle and tumor-to-liver ratios. Reproduced with permission from ref. [47]. Copyright 2024 American Chemical Society. (**b**) MSN@CUR suppresses tumor growth in H22 subcutaneous models, as evidenced by reduced tumor size, volume, and weight; altered microvessel morphology; altered H&E histology; and modulation of VEGF, AKT, p-PI3K, caspase-3, and caspase-8. A schematic of the proposed liver cancer therapeutic mechanism is shown. Reproduced with permission from ref. [48]. Copyright 2024 American Chemical Society. (**c**) Bioconjugation strategy for multifunctional mesoporous silica nanoparticles. Reproduced with permission from ref. [49]. Copyright 2020 American Chemical Society.

Silica-based nanoplatforms have gained increasing attention for cancer theranostics and targeted therapy owing to their structural tunability, favorable pharmacokinetics, and multifunctionality. Ultrasmall porous silica nanoparticles (UPSNs) conjugated with the isotopic pair yttrium-86/yttrium-90 (^86^Y/^90^Y) demonstrated prolonged blood circulation, efficient evasion of the reticuloendothelial system, and high tumor accumulation (~12% injected dose per gram), enabling both positron emission tomography imaging and effective internal radiotherapy; notably, ^90^Y-DOTA-UPSN significantly inhibited tumor growth and improved survival with minimal off-target toxicity (Figure 8a) [47]. In parallel, lactobionic acid–modified carboxymethyl chitosan–coated mesoporous silica nanoparticles (LA-CMCS-MSN@CUR) were developed to enhance the bioavailability and tumor targeting of curcumin (CUR) for hepatocellular carcinoma, achieving receptor-mediated uptake, pH-responsive drug release, and superior antitumor efficacy via suppression of VEGF/PI3K/AKT signaling and activation of apoptotic pathways (Figure 8b) [48]. Furthermore, transferrin-functionalized mesoporous silica nanoparticles (MSNPs) chelated with 3,4,3-LI(1,2-HOPO) enabled stable loading of the alpha emitter actinium-225 (^225^Ac), resulting in efficient breast cancer cell targeting, potent cytotoxicity, enhanced radionuclide clearance, and minimal bone accumulation in vivo (Figure 8c) [49].

### 3.2. PEG-Based Nanoparticles for Targeted Cancer Treatment

**Figure 9:**
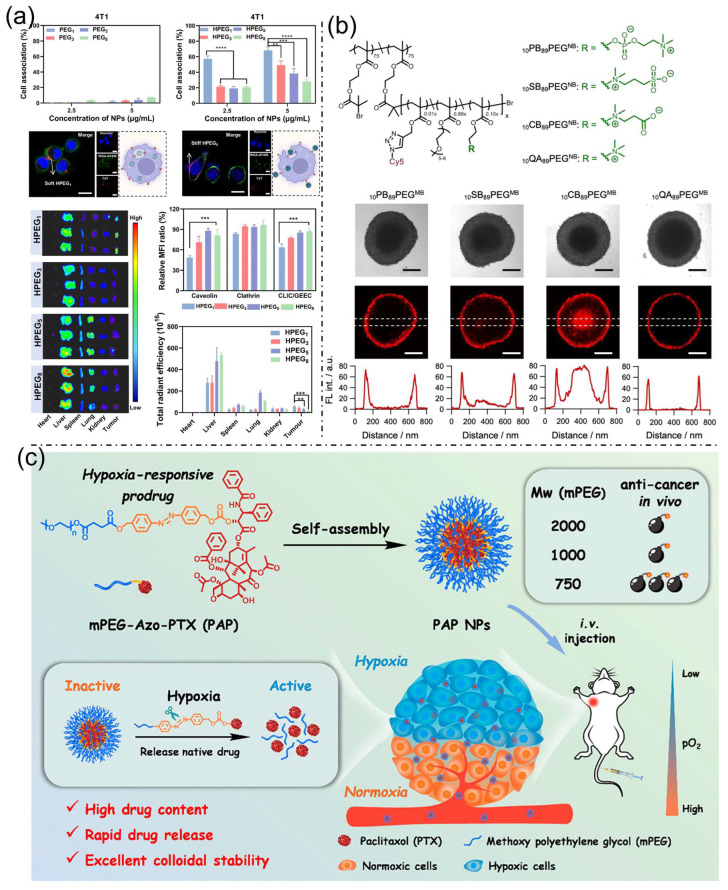
(**a**) Cellular uptake of PEG and HPEG nanoparticles in 4T1 cells after 12 h, shown by flow cytometry and confocal microscopy with nuclear/membrane staining, and in vivo fluorescence imaging of HPEG distribution at 12 h, including quantitative analysis of endocytic inhibitor effects on uptake and organ distribution. Reproduced with permission from ref. [50]. Copyright 2025 American Chemical Society. (**b**). Molecular architectures of microbubbles (MBs) consisting of 89 mol% pEGMA combined with 10 mol% betaine-based polymers (pPBMA, pSBMA, or pCBMA) or cationic polymer (pQAMA). Bright-field images (top), confocal laser scanning microscopy (CLSM) images captured near the mid-plane of the spheroids (middle), and corresponding fluorescence intensity profiles (bottom) of CT26 tumor spheroids after 3 h incubation with Cy5-tagged MBs in culture medium are shown. Panels represent spheroids treated with _10_PB_89_PEG^MB^, _10_SB_89_PEG^MB^, _10_CB_89_PEG^MB^, and _10_QA_89_PEG^MB^. Red fluorescence denotes the distribution of MBs within the spheroids. Reproduced with permission from ref. [51]. Copyright 2022 American Chemical Society. (**c**) Schematic illustration of the self-assembly behavior, hypoxia-responsive drug release, and antitumor efficacy of PEGylated PTX prodrugs. Reproduced with permission from ref. [52]. Copyright 2022 American Chemical Society.

Nanoparticle design parameters such as mechanical stiffness, surface chemistry, and stimuli-responsive drug release play crucial roles in determining the in vivo performance of nanocarriers for cancer therapy. Modulating nanoparticle stiffness via a layer-by-layer assembly of poly(ethylene glycol) (PEG) nanoparticles demonstrated that softer particles form less protein corona, exhibit prolonged blood circulation, reduced liver accumulation, and, when functionalized with hyaluronic acid, achieve enhanced cellular targeting, tumor accumulation, and tumor growth inhibition in vivo (Figure 9a) [50]. Beyond stiffness, polymer chemistry significantly influences tumor penetration. Meanwhile, conventional PEG-based molecular bottlebrushes showed limited cellular interaction and tumor infiltration, zwitterionic bottlebrushes—particularly those incorporating carboxybetaine moieties—enabled deep tumor penetration, efficient cellular uptake, and transcytosis-mediated transport, with even low zwitterionic content markedly improving PEG-based systems (Figure 9b) [51]. Additionally, controlled and tumor-specific drug release was achieved through hypoxia-responsive PEG–paclitaxel prodrug nanoparticles employing azobenzene linkers, which self-assembled into high–drug-loading nanoparticles and selectively released paclitaxel in hypoxic tumor environments, resulting in superior antitumor efficacy and reduced systemic toxicity, especially for formulations with shorter PEG chains (Figure 9c) [52].

### 3.3. Graphene Oxide-Based Nanoplatforms in Cancer Therapy

**Figure 10:**
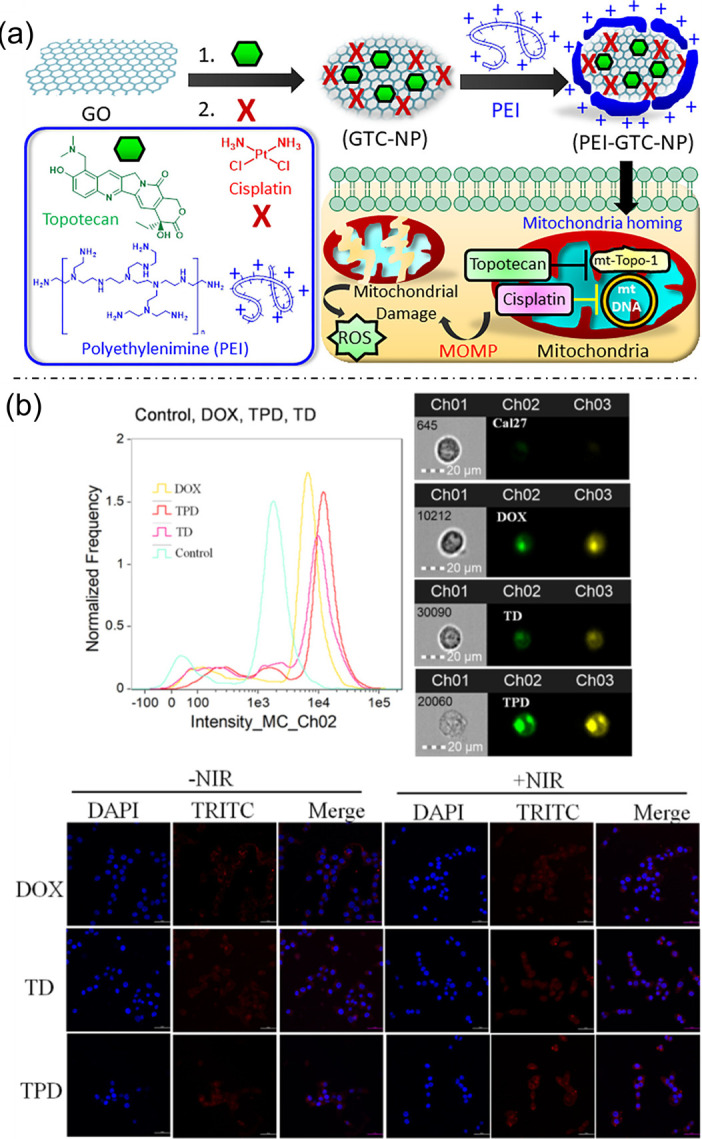
(**a**) Engineering of polyethylenimine-coated graphene oxide nanoparticles encapsulating topotecan and cisplatin (PEI-GTC-NP) for mitochondria-targeted cancer therapy. Reproduced with permission from ref. [53]. Copyright 2019 American Chemical Society. (**b**) Cellular internalization of free DOX, TD, and TPD in CAL-27 cells at equivalent DOX concentration, shown by flow cytometry, fluorescence imaging, and confocal microscopy with/without NIR irradiation, illustrating DOX uptake and intracellular distribution (DAPI-stained nuclei). Reproduced with permission from ref. [54]. Copyright 2020 American Chemical Society.

Graphene oxide–based nanoplatforms have emerged as versatile tools for cancer therapy owing to their unique physicochemical properties and adaptability for multifunctional design. Mitochondria, which regulate cellular bioenergetics, metabolism, and signaling, represent an attractive yet challenging subcellular target; to address this, polyethylenimine-coated self-assembled graphene oxide nanoparticles (PEI-GTC-NPs) were engineered to co-deliver topotecan and cisplatin, achieving rapid mitochondrial accumulation in HeLa cervical cancer cells and inducing mitochondrial membrane disruption, excessive reactive oxygen species generation, and enhanced cancer cell death (Figure 10a) [53]. Beyond subcellular targeting, graphene oxide has also been exploited for synergistic therapeutic strategies. Tea polyphenol–reduced and functionalized graphene oxide (TPG) conjugated with anti–programmed death ligand 1 antibodies and loaded with doxorubicin enabled targeted chemo–photothermal therapy, where near-infrared irradiation triggered efficient photothermal conversion and pH-responsive drug release, resulting in significantly enhanced apoptosis in PD-L1–overexpressing CAL27 tongue squamous carcinoma cells with reduced toxicity to normal cells (Figure 10b) [54]. In addition, tumor microenvironment–responsive drug delivery was achieved using graphene oxide nanoparticles (GONs) coated with chitosan and dimethylmaleic anhydride–modified chitosan (CS/CS-DMMA), where pH-triggered charge reversal promoted enhanced cellular uptake and site-specific release of doxorubicin, leading to improved therapeutic efficacy in HepG2 cancer cells [55].

**Table 2:** Advanced Polymeric Nanoparticle Nanoplatforms for Cancer Theragnostics.

Polymeric Nanoplatform System	Imaging Modality/Therapeutic Agent	Targeting Strategy/Cancer Type	Key Features	Ref.
Hybrid PLGA-Phospholipid Nanoparticles (PLGA core with mPEG-DSPE shell)	Imaging: MRI (Gd^3+^-DOTA chelates), Fluorescence (Rhodamine 6G) Therapy: Paclitaxel	Targeting: Passive (EPR effect), PEGylation for stealth Cancer: Pancreatic cancer (PANC-1, MIA PaCa-2)	Size-tunable (65–110 nm), multimodal theragnostic platform with controlled drug release demonstrated superior tumor retention in vivo.	[56]
PEG-PLGA Nanoparticles with EpCAM Aptamer	Imaging: Not specified Therapy: Doxorubicin (DOX)	Targeting: EpCAM aptamer for active targeting Cancer: Breast cancer	Enhanced cytotoxicity and tumor inhibition compared to unmodified nanoparticles, targeting EpCAM-overexpressing cancer cells.	[57]
Chitosan Nanoparticles with Folic Acid and TPGS-SH (Dual-receptor targeted)	Imaging: Photoacoustic imaging, IVIS fluorescence Therapy: Cabazitaxel (CZT)	Targeting: Folic acid (FR) + Cetuximab (EGFR) dual targeting, pH-responsive Cancer: Lung cancer (A549 cells)	Redox-sensitive TPGS-SH moiety, 6.3-fold enhanced cytotoxicity (IC_50_ = 0.26 µg/mL), improved lung targeting.	[58]
Glycol-Chitosan Gold Nanoparticles	Imaging: Photoacoustic imaging Therapy: Tumor antigen delivery for immunotherapy	Targeting: Passive lymph node targeting Cancer: Cancer immunotherapy (OVA model)	No targeting ligand is required for lymph node accumulation, which delivers antigens to macrophages for an immune response.	[59]
PAMAM Dendrimer with SPIO and Folic Acid	Imaging: MRI (T_2_-weighted, Fe_3_O_4_), Fluorescence (Cy5.5) Therapy: Paclitaxel	Targeting: Folic acid for FR-positive cells Cancer: Breast cancer (MCF-7), Hepatocarcinoma (H22)	pH-responsive ester bond for controlled release, G3.5 PAMAM generation, multimodal imaging capability.	[60]
Polymeric Micelles (PEG-PCL) with Tranilast	Imaging: Shear wave elastography (SWE) for stiffness monitoring Therapy: Tranilast (TME remodeling) + Epirubicin or Doxil	Targeting: TME normalization, combined with immunotherapy Cancer: Triple-negative breast cancer (4T1, E0771)	Reprogramming tumor microenvironment, enhancing T-cell infiltration, 100× lower dose than free drug, SWE predicts response.	[61]
Hyaluronic Acid-PLGA Block Copolymer (HAssLG with disulfide linkage)	Imaging: NIR fluorescence imaging Therapy: Doxorubicin	Targeting: CD44 receptor-mediated endocytosis Cancer: Breast cancer (MDA-MB231)	Redox-responsive (GSH-cleavable), specific delivery to CD44-positive tumors, dual targeting via EPR and CD44 receptor.	[62]
Albumin-Templated CuS Nanoparticles	Imaging: NIR fluorescence, Photoacoustic, MRI (Gd-DTPA) Therapy: Photothermal therapy (PTT)	Targeting: Passive (EPR effect), optional peptide conjugation Cancer: Various (breast cancer 4T1, glioblastoma)	Trimodal NIRF/PA/MR imaging, strong NIR absorption for PTT, complete tumor elimination without regrowth.	[63]
Gelatin Nanoparticles with EGFR Peptide	Imaging: Not specified Therapy: Gemcitabine	Targeting: EGFR-targeting peptide, redox-responsive Cancer: Pancreatic adenocarcinoma (Panc-1 orthotopic)	PEGylated for circulation, disulfide bond cleavage for drug release, and superior antitumor activity in the orthotopic model.	[64]
Quantum Dot-Mesoporous Silica-Gold Hybrid (QD@MSN-Au with aptamer)	Imaging: Fluorescence (GZCIS/ZnS QDs), MRI (magnetic QDs) Therapy: Epirubicin	Targeting: EpCAM aptamers for active targeting Cancer: Colorectal cancer (CRC cells)	Multifunctional platform: QDs for imaging, MSN for drug loading, Au NPs for capping, aptamer-targeted, effective tumor suppression.	[65]

## 4. Biocompatibility, Safety, and Regulatory Perspectives

Polymeric and metallic nanoparticles exhibit fundamentally distinct biocompatibility and safety profiles, requiring tailored regulatory strategies. Polymeric nanoparticles, composed of biodegradable materials like poly(lactic-co-glycolic acid) (PLGA) and polyethylene glycol (PEG), generally exhibit superior biocompatibility due to their capacity to degrade into non-toxic byproducts [66,67,68,69,70]. However, immunogenicity remains a critical concern—even degraded polymeric fragments can trigger unintended inflammatory responses, necessitating rigorous assessment of immune activation and long-term organ accumulation patterns. The FDA-approved polymeric nanoparticle therapies, including PLGA-based formulations such as Lupron Depot, exemplify successful translation, yet their regulatory pathways require extensive toxicological characterization. Conversely, metallic nanoparticles—gold, silver, copper oxide, and zinc oxide variants—pose inherent toxicity risks driven by size-dependent effects and metal ion dissolution. Particles smaller than 20 nanometers exhibit enhanced cellular penetration and elevated reactive oxygen species (ROS) generation, leading to oxidative stress, genotoxicity, and potential mitochondrial dysfunction. Surface functionalization with biocompatible polymers significantly mitigates these risks; antibody-coated, starch-dextran–functionalized, and PEGylated metallic nanoparticles exhibit substantially reduced cytotoxicity compared to bare counterparts [66,67,68,69,70].

The FDA and the European Medicines Agency (EMA) have implemented adaptive regulatory frameworks to address nanomaterial-specific challenges, evaluating each product on a case-by-case basis under existing statutory authorities. Current guidance documents emphasize comprehensive nanocharacterization, including physicochemical profiling, biodistribution mapping, and long-term safety assessment [67,71]. The FDA’s Nanotechnology Characterization Laboratory (NCL), along with international collaborations through the Innovation Task Force, supports standardized preclinical testing protocols; however, gaps in early regulatory engagement often delay approvals. Polymeric nanoparticles require detailed evaluation of polymer degradation kinetics and metabolite clearance, while metallic nanoparticles necessitate dose-escalation studies to assess organ accumulation and chronic exposure effects. Recent advances, such as AI-driven biocompatibility predictions and the repurposing of FDA-approved excipients to reduce immunogenicity, offer promising mitigation strategies. Nevertheless, challenges related to manufacturing reproducibility, scale-up consistency, and inter-patient variability persist, highlighting the need for robust quality-by-design approaches and harmonized bioanalytical standards across regulatory jurisdictions. Incorporating patient stratification biomarkers, imaging-based monitoring of nanoparticle accumulation, and adaptive clinical trial designs is expected to accelerate the regulatory approval of nanomedicine platforms in the future.

## 5. Challenges and Future Perspectives for Polymeric and Metallic Nanoparticles in Cancer Theragnostics

### 5.1. Current Challenges

Clinical translation of polymeric nanoparticles remains severely limited despite promising preclinical results. Key barriers include immunogenicity, where organic polymers and their hybrid counterparts can trigger inflammatory responses [72,73]. Pharmacokinetic variability across patient populations complicates dose optimization and therapeutic predictability. The enhanced permeability and retention (EPR) effect, long considered fundamental to passive tumor accumulation, exhibits highly variable efficacy and significant heterogeneity among patients [73]. Manufacturing scalability poses critical constraints—maintaining consistent quality, reproducibility, and batch-to-batch uniformity at large scale remains unresolved. Long-term safety concerns regarding organ accumulation and chronic toxicity from non-biodegradable polymer byproducts present additional regulatory barriers. Metallic nanoparticles face distinct toxicity challenges driven by their nanoscale properties. Size-dependent effects are profound—particles of 10 nm penetrate biological membranes more readily than larger particles, thereby increasing oxidative stress and reactive oxygen species (ROS) [66,74]. Metal ion dissolution in physiological environments poses dose-dependent toxicity risks. Immunotoxicity, nephrotoxicity, and neurotoxicity represent documented side effects. Surface functionalization with biopolymers improves biocompatibility but adds manufacturing complexity and potential immunogenic reactions. Additionally, immune system interactions and potential immunogenicity of certain materials can compromise therapeutic outcomes. The integration of multiple imaging and therapeutic modalities introduces further complexity, requiring careful optimization to balance efficacy, safety, and stability. Finally, regulatory hurdles and standardization across different jurisdictions present significant barriers to rapid clinical translation, particularly for novel hybrid or multifunctional nanoplatforms. Addressing these challenges is critical for realizing the full potential of nanomedicine in personalized, image-guided cancer therapy.

### 5.2. Future Perspectives

The rapid progress in multifunctional nanoplatforms offers a transformative path for cancer diagnosis and therapy, yet several avenues remain for further exploration. Future research should focus on enhancing the biocompatibility and long-term stability of nanoparticles to minimize immune responses and off-target effects [75,76]. Scalable and reproducible manufacturing strategies are essential to facilitate clinical translation from preclinical studies [77]. Additionally, personalized nanomedicine approaches—tailoring nanoparticle design to tumor-specific microenvironments, such as hypoxia, acidic pH, and enzyme expression—could further improve targeting precision and therapeutic efficacy [78]. The integration of advanced imaging modalities with real-time therapeutic monitoring will enable adaptive, image-guided treatments, optimizing outcomes on an individual basis. Finally, multimodal and hybrid nanoplatforms that combine drug delivery with photothermal, photodynamic, or sonodynamic therapies hold significant promise for synergistic, minimally invasive cancer treatment [79]. Addressing these challenges through interdisciplinary collaboration will accelerate the translation of nanotechnology-based theranostics into routine clinical oncology, ultimately enhancing treatment precision, reducing side effects, and improving patient survival.

## 6. Conclusions

The convergence of multifunctional nanomaterials with advanced imaging and therapeutic strategies marks a pivotal advancement in cancer diagnosis and treatment. Nanoplatforms, including gold, iron oxide, silver, titanium-based systems, and polymeric nanoparticles (PNPs), offer distinct physicochemical properties that can be tailored for enhanced tumor targeting, multimodal imaging, and effective therapeutic delivery. These innovations enable precise tumor localization and real-time monitoring of treatment responses. By exploiting the tumor microenvironment’s unique characteristics, such as acidic pH, hypoxia, and enzymatic overexpression, and utilizing cutting-edge synthetic and surface-engineering approaches, these nanoplatforms achieve superior imaging sensitivity, controlled drug release, and improved therapeutic efficacy, all while minimizing off-target toxicity. The integration of diagnostic and therapeutic functions into a single nanoplatform facilitates personalized, image-guided cancer theranostics, allowing for individualized treatment planning and adaptive therapy. These developments underscore the transformative potential of engineered nanoplatforms in bridging the gap between preclinical research and clinical applications, offering prospects for improved patient outcomes and accelerated translation of nanomedicine into routine oncology practice. Future research should focus on optimizing the properties of these nanoparticles to overcome challenges such as scale-up production, immune responses, and long-term stability. Continued innovation in nanoparticle design holds immense promise for enhancing therapeutic efficacy, reducing side effects, and ultimately advancing cancer therapies in clinical settings.

## Data Availability

The original contributions presented in the study are included in the article, further inquiries can be directed to the corresponding author.

## References

[1] Hamidu A., Pitt W.G., Husseini G.A. (2023). Recent Breakthroughs in Using Quantum Dots for Cancer Imaging and Drug Delivery Purposes. Nanomaterials.

[2] Roshani M., Rezaian-Isfahni A., Lotfalizadeh M.H., Khassafi N., Abadi M.H.J.N., Nejati M. (2023). Metal nanoparticles as a potential technique for the diagnosis and treatment of gastrointestinal cancer: A comprehensive review. Cancer Cell Int..

[3] Chatterjee P., Kumar S. (2022). Current developments in nanotechnology for cancer treatment. Mater. Today Proc..

[4] Păduraru D.N., Ion D., Niculescu A.-G., Mușat F., Andronic O., Grumezescu A.M., Bolocan A. (2022). Recent developments in metallic nanomaterials for cancer therapy, diagnosing and imaging applications. Pharmaceutics.

[5] Yang Z., Xu T., Li H., She M., Chen J., Wang Z., Zhang S., Li J. (2023). Zero-dimensional carbon nanomaterials for fluorescent sensing and imaging. Chem. Rev..

[6] Haijia Y., Jianhua L., Wei L., Rui N., Bin Z., Yuan X., Yang L., Yinghui W., Hongjie Z. (2025). Metal-based nanomedicines for cancer theranostics. Mil. Med. Res..

[7] Khorasani A., Shahbazi-Gahrouei D., Safari A. (2023). Recent metal nanotheranostics for cancer diagnosis and therapy: A review. Diagnostics.

[8] Kumar D., Mutreja I., Kaushik A. (2023). Recent advances in noble metal nanoparticles for cancer nanotheranostics. J. Nanotheranostics.

[9] Figueiredo A.Q., Rodrigues C.F., Fernandes N., de Melo-Diogo D., Correia I.J., Moreira A.F. (2022). Metal-polymer nanoconjugates application in cancer imaging and therapy. Nanomaterials.

[10] Heying L., Shaowen C., Jingming Z., Kun L., Kaiyong C., Jinghua L. (2023). Platinum based theranostics nanoplatforms for antitumor applications. J. Mater. Chem. B.

[11] Niu G., Gao F., Wang Y., Zhang J., Zhao L., Jiang Y. (2022). Bimetallic nanomaterials: A promising nanoplatform for multimodal cancer therapy. Molecules.

[12] Donald A.F. (2023). Review on Metal-Based Theranostic Nanoparticles for Cancer Therapy and Imaging. Technol. Cancer Res. Treat..

[13] Subhan M.A., Muzibur Rahman M. (2022). Recent development in metallic nanoparticles for breast cancer therapy and diagnosis. Chem. Rec..

[14] Moore J.A., Chow J.C. (2021). Recent progress and applications of gold nanotechnology in medical biophysics using artificial intelligence and mathematical modeling. Nano Express.

[15] Liu Y., Li J., Chen M., Chen X., Zheng N. (2020). Palladium-based nanomaterials for cancer imaging and therapy. Theranostics.

[16] Cheng X., Zhou X., Xu J., Sun R., Xia H., Ding J., Chin Y.E., Chai Z., Shi H., Gao M. (2021). Furin Enzyme and pH Synergistically Triggered Aggregation of Gold Nanoparticles for Activated Photoacoustic Imaging and Photothermal Therapy of Tumors. Anal. Chem..

[17] Liu A., Li L., Wang Z., Li X., Liang H., Yang J., Nesic M.D., Yang X., Lin Q. (2025). Ultrasmall Au-GRHa Nanosystem for FL/CT Dual-Mode Imaging-Guided Targeting Photothermal Therapy of Ovarian Cancer. Anal. Chem..

[18] Zhang L., Chen S., Ma R., Zhu L., Yan T., Alimu G., Du Z., Alifu N., Zhang X. (2021). NIR-excitable PEG-modified Au nanorods for photothermal therapy of cervical cancer. ACS Appl. Nano Mater..

[19] Shi X.-H., Zhao W., Yang Z., Wang L., Ren W. (2025). Simultaneous Guidance of Intraoperative Tumor Resection by Near-Infrared-II Imaging Combined with Complementary Surface-Enhanced Raman Imaging via Janus Au-PbS Nanoparticles. Anal. Chem..

[20] Huang Y., Li J., Feng H., Du H., Deng Z. (2025). A rapidly synthesized, ultrasmall silver nanocluster for near-infrared-II imaging and metabolic studies. Nano Lett..

[21] Zhu S., Song W., He Y., Wang Y., Li X., Wu Y., Meng X., Lin C., Wang W., Wang H. (2025). Biosynthetic Ag2S Nanoparticles for Photoacoustic Imaging-Guided Photothermal Therapy. ACS Appl. Mater. Interfaces.

[22] Xie M., Li Y., Xu Y., Zhang Z., Ji B., Jones J.B., Wang Z., Mao H. (2022). Brain tumor imaging and delivery of sub-5 nm magnetic iron oxide nanoparticles in an orthotopic murine model of glioblastoma. ACS Appl. Nano Mater..

[23] Cao Y., Mao Z., He Y., Kuang Y., Liu M., Zhou Y., Zhang Y., Pei R. (2020). Extremely small iron oxide nanoparticle-encapsulated nanogels as a glutathione-responsive T1 contrast agent for tumor-targeted magnetic resonance imaging. ACS Appl. Mater. Interfaces.

[24] Zhang L., Tong S., Zhang Q., Bao G. (2020). Lipid-encapsulated Fe3O4 nanoparticles for multimodal magnetic resonance/fluorescence imaging. ACS Appl. Nano Mater..

[25] Sun X., Tan M., Fang J., Wang S., Guo Y., Cao Z., Xie T., Xu K., Zhao Z., Zhang W. (2024). Cubic Zinc-Doped Iron Oxide Nanoparticles with Poly (Ethylene Glycol) or Sodium Citrate Surface Coatings for Tumor Imaging. ACS Appl. Nano Mater..

[26] Bai S., Yang N., Wang X., Gong F., Dong Z., Gong Y., Liu Z., Cheng L. (2020). Ultrasmall Iron-Doped Titanium Oxide Nanodots for Enhanced Sonodynamic and Chemodynamic Cancer Therapy. ACS Nano.

[27] Xie Z., Chen S., Duo Y., Zhu Y., Fan T., Zou Q., Qu M., Lin Z., Zhao J., Li Y. (2019). Biocompatible two-dimensional titanium nanosheets for multimodal imaging-guided cancer theranostics. ACS Appl. Mater. Interfaces.

[28] Li J., Dai S., Qin R., Shi C., Ming J., Zeng X., Wen X., Zhuang R., Chen X., Guo Z. (2021). Ligand engineering of titanium-oxo nanoclusters for Cerenkov radiation-reinforced photo/chemodynamic tumor therapy. ACS Appl. Mater. Interfaces.

[29] Shi Z., Zhang K., Zada S., Zhang C., Meng X., Yang Z., Dong H. (2020). Upconversion nanoparticle-induced multimode photodynamic therapy based on a metal–organic framework/titanium dioxide nanocomposite. ACS Appl. Mater. Interfaces.

[30] Mansoorianfar M., Hussain Z., Simchi A., Cao Y., Ullah I., Ullah S. (2023). Target-responsive DNA aptamer-conjugated superparamagnetic Ag/CuS nanoparticles as near-infrared light-triggered theranostics and dual-modal imaging. Appl. Mater. Today.

[31] Yang Y., Zheng W., Zhang J., Guo J., Liu Q., Wang H., Xu F., Bao Z. (2025). Integrating Photothermal, Photodynamic, and Chemodynamic Therapies: The Innovative Design Based on Copper Sulfide Nanoparticles for Enhanced Tumor Therapy. ACS Appl. Bio Mater..

[32] Qiang S., Hu X., Li R., Wu W., Fang K., Li H., Sun Y., Liang S., Zhao W., Wang M. (2022). CuS Nanoparticles-Loaded and Cisplatin Prodrug Conjugated Fe(III)–MOFs for MRI-Guided Combination of Chemotherapy and NIR-II Photothermal Therapy. ACS Appl. Mater. Interfaces.

[33] Lv H., Zhu Y., Xue J., Jia X., Chen J. (2022). Targeted Drug Delivery System Based on Copper Sulfide for Synergistic Near-Infrared Photothermal Therapy/Photodynamic Therapy/Chemotherapy of Triple Negative Breast Cancer. Langmuir.

[34] Zhu Y., Wang Z., Zhao R., Zhou Y., Feng L., Gai S., Yang P. (2022). Pt Decorated Ti3C2Tx MXene with NIR-II Light Amplified Nanozyme Catalytic Activity for Efficient Phototheranostics. ACS Nano.

[35] Li Z.-H., Chen Y., Sun Y., Zhang X.-Z. (2021). Platinum-Doped Prussian Blue Nanozymes for Multiwavelength Bioimaging Guided Photothermal Therapy of Tumor and Anti-Inflammation. ACS Nano.

[36] Zhang Q., Lin J., Li J., Zhou Y., Bi Z., Yang H., Lu W., Lu T., Qian R., Yang X. (2025). Mitochondrial-Targeted Multifunctional Platinum-Based Nano “Terminal-Sensitive Projectile” for Enhanced Cancer Chemotherapy Efficacy. ACS Nano.

[37] Luo Y., Zheng F., Gao Y., Chen W., Xue X., Xiao C., Wei K. (2024). Flower-Like MnO2 Nanoparticle-Based Dual GSH-Depleting Nanosystems for Chemo-/Chemodynamic/Sonodynamic Cancer Therapy. ACS Appl. Nano Mater..

[38] Wu Q., Tan L., Ren X., Fu C., Chen Z., Ren J., Ma T., Meng X. (2023). Metal–Organic Framework-Based Nano-Activators Facilitating Microwave Combined Therapy via a Divide-and-Conquer Tactic for Triple-Negative Breast Cancer. ACS Nano.

[39] Li J., Qu B., Wang Q., Ning X., Ren S., Liu C., Zhang R. (2022). Hollow Manganese-Doped Calcium Phosphate Nanoparticles Treated with Melanin Nanoparticles and Thalidomide for Photothermal and Anti-angiogenic Cancer Therapy. ACS Appl. Nano Mater..

[40] Ying-Ying M., Jing M., Haojie Q., Pan L., Wenpeng H., Chenchen L., Jianbo G. (2022). Nano-Metal–Organic Framework Decorated With Pt Nanoparticles as an Efficient Theranostic Nanoprobe for CT/MRI/PAI Imaging-Guided Radio-Photothermal Synergistic Cancer Therapy. Front. Bioeng. Biotechnol..

[41] Sichao T., Qian Z., Zhi H., Weidong Z., Zhuo A., Dong H., Qing-Hua X. (2024). A “Transformers”-like nanochain for precise navigation and efficient cancer treatment. Aggregate.

[42] Shun L., Shuijie Q., Gerile O., Li Z. (2022). Iron-Based Hollow Nanoplatforms for Cancer Imaging and Theranostics. Nanomaterials.

[43] Austria E., Bilek M., Varamini P., Akhavan B. (2025). Breaking biological barriers: Engineering polymeric nanoparticles for cancer therapy. Nano Today.

[44] Hosseini S.M., Mohammadnejad J., Salamat S., Beiram Zadeh Z., Tanhaei M., Ramakrishna S. (2023). Theranostic polymeric nanoparticles as a new approach in cancer therapy and diagnosis: A review. Mater. Today Chem..

[45] Xiao X., Teng F., Shi C., Chen J., Wu S., Wang B., Meng X., Essiet Imeh A., Li W. (2022). Polymeric nanoparticles—Promising carriers for cancer therapy. Front. Bioeng. Biotechnol..

[46] Madej M., Kurowska N., Strzalka-Mrozik B. (2022). Polymeric Nanoparticles—Tools in a Drug Delivery System in Selected Cancer Therapies. Appl. Sci..

[47] Ferreira C.A., Goel S., Ehlerding E.B., Rosenkrans Z.T., Jiang D., Sun T., Aluicio-Sarduy E., Engle J.W., Ni D., Cai W. (2021). Ultrasmall Porous Silica Nanoparticles with Enhanced Pharmacokinetics for Cancer Theranostics. Nano Lett..

[48] Man S., Liu W., Bi J., Bai J., Wu Q., Hu B., Hu J., Ma L. (2024). Smart Mesoporous Silica Nanoparticles Loading Curcumin Inhibit Liver Cancer. J. Agric. Food Chem..

[49] Pallares R.M., Agbo P., Liu X., An D.D., Gauny S.S., Zeltmann S.E., Minor A.M., Abergel R.J. (2020). Engineering Mesoporous Silica Nanoparticles for Targeted Alpha Therapy against Breast Cancer. ACS Appl. Mater. Interfaces.

[50] Li M., Gao Z., Lv H., Sekhar K.P.C., Song A., Jiang X., Hao J., Cui J. (2025). Multilayered Nanoarchitectonics of Poly(ethylene glycol) Nanoparticles with Tunable Stiffness Modulate Bio–Nano Interactions and Targeted Drug Delivery. ACS Nano.

[51] Fujii S., Takano S., Nakazawa K., Sakurai K. (2022). Impact of Zwitterionic Polymers on the Tumor Permeability of Molecular Bottlebrush-Based Nanoparticles. Biomacromolecules.

[52] Hao D., Meng Q., Jiang B., Lu S., Xiang X., Pei Q., Yu H., Jing X., Xie Z. (2022). Hypoxia-Activated PEGylated Paclitaxel Prodrug Nanoparticles for Potentiated Chemotherapy. ACS Nano.

[53] Mallick A., Nandi A., Basu S. (2019). Polyethylenimine Coated Graphene Oxide Nanoparticles for Targeting Mitochondria in Cancer Cells. ACS Appl. Bio Mater..

[54] Hao L., Song H., Zhan Z., Lv Y. (2020). Multifunctional Reduced Graphene Oxide-Based Nanoplatform for Synergistic Targeted Chemo-Photothermal Therapy. ACS Appl. Bio Mater..

[55] Zhao X., Wei Z., Zhao Z., Miao Y., Qiu Y., Yang W., Jia X., Liu Z., Hou H. (2018). Design and Development of Graphene Oxide Nanoparticle/Chitosan Hybrids Showing pH-Sensitive Surface Charge-Reversible Ability for Efficient Intracellular Doxorubicin Delivery. ACS Appl. Mater. Interfaces.

[56] Tng D.J., Song P., Lin G., Soehartono A.M., Yang G., Yang C., Yin F., Tan C.H., Yong K.T. (2015). Synthesis and characterization of multifunctional hybrid-polymeric nanoparticles for drug delivery and multimodal imaging of cancer. Int. J. Nanomed..

[57] Nath V., Singh M., Jana B.K., Sarkar T., Gogoi N.R., Mazumder B. (2024). PLGA and cancer: A comprehensive patent-based review on the present state of art. Pharm. Pat. Anal..

[58] Vikas, Mehata A.K., Viswanadh M.K., Malik A.K., Setia A., Kumari P., Mahto S.K., Muthu M.S. (2023). EGFR Targeted Redox Sensitive Chitosan Nanoparticles of Cabazitaxel: Dual-Targeted Cancer Therapy, Lung Distribution, and Targeting Studies by Photoacoustic and Optical Imaging. Biomacromolecules.

[59] Sun I.C., Jo S., Dumani D., Yun W.S., Yoon H.Y., Lim D.K., Ahn C.H., Emelianov S., Kim K. (2021). Theragnostic Glycol Chitosan-Conjugated Gold Nanoparticles for Photoacoustic Imaging of Regional Lymph Nodes and Delivering Tumor Antigen to Lymph Nodes. Nanomaterials.

[60] Ray S., Li Z., Hsu C.H., Hwang L.P., Lin Y.C., Chou P.T., Lin Y.Y. (2018). Dendrimer- and copolymer-based nanoparticles for magnetic resonance cancer theranostics. Theranostics.

[61] Panagi M., Mpekris F., Chen P., Voutouri C., Nakagawa Y., Martin J.D., Hiroi T., Hashimoto H., Demetriou P., Pierides C. (2022). Polymeric micelles effectively reprogram the tumor microenvironment to potentiate nano-immunotherapy in mouse breast cancer models. Nat. Commun..

[62] Park H.K., Lee S.J., Oh J.S., Lee S.G., Jeong Y.I., Lee H.C. (2015). Smart Nanoparticles Based on Hyaluronic Acid for Redox-Responsive and CD44 Receptor-Mediated Targeting of Tumor. Nanoscale Res. Lett..

[63] An F.-F., Zhang X.-H. (2017). Strategies for Preparing Albumin-based Nanoparticles for Multifunctional Bioimaging and Drug Delivery. Theranostics.

[64] Singh A., Xu J., Mattheolabakis G., Amiji M. (2016). EGFR-targeted gelatin nanoparticles for systemic administration of gemcitabine in an orthotopic pancreatic cancer model. Nanomedicine.

[65] Abrishami A., Bahrami A.R., Nekooei S., Saljooghi A.S., Matin M.M. (2024). Hybridized quantum dot, silica, and gold nanoparticles for targeted chemo-radiotherapy in colorectal cancer theranostics. Commun. Biol..

[66] Asghar M.A., Yousuf R.I., Shoaib M.H., Asghar M.A., Mumtaz N. (2021). A Review on Toxicity and Challenges in Transferability of Surface-functionalized Metallic Nanoparticles from Animal Models to Humans. BIO Integr..

[67] Eltaib L. (2025). Polymeric Nanoparticles in Targeted Drug Delivery: Unveiling the Impact of Polymer Characterization and Fabrication. Polymers.

[68] Zhang N., Xiong G., Liu Z. (2022). Toxicity of metal-based nanoparticles: Challenges in the nano era. Front. Bioeng. Biotechnol..

[69] Gao F., Feng X., Li X. (2025). Recent advances in polymeric nanoparticles for the treatment of hepatic diseases. Front. Pharmacol..

[70] Egbuna C., Parmar V.K., Jeevanandam J., Ezzat S.M., Patrick-Iwuanyanwu K.C., Adetunji C.O., Khan J., Onyeike E.N., Uche C.Z., Akram M. (2021). Toxicity of Nanoparticles in Biomedical Application: Nanotoxicology. J. Toxicol..

[71] Rodríguez F., Caruana P., De la Fuente N., Español P., Gámez M., Balart J., Llurba E., Rovira R., Ruiz R., Martín-Lorente C. (2022). Nano-Based Approved Pharmaceuticals for Cancer Treatment: Present and Future Challenges. Biomolecules.

[72] Shanahan K., Coen D., Nafo W. (2025). Polymer-based nanoparticles for cancer theranostics: Advances, challenges, and future perspectives. Explor. BioMat-X.

[73] Li Y., He J., Liu J., Um W., Ding J. (2024). Challenges and opportunities of poly(amino acid) nanomedicines in cancer therapy. Nanomedicine.

[74] Attarilar S., Yang J., Ebrahimi M., Wang Q., Liu J., Tang Y., Yang J. (2020). The Toxicity Phenomenon and the Related Occurrence in Metal and Metal Oxide Nanoparticles: A Brief Review From the Biomedical Perspective. Front. Bioeng. Biotechnol..

[75] Ammar M.M., Ali R., Abd Elaziz N.A., Habib H., Abbas F.M., Yassin M.T., Maniah K., Abdelaziz R. (2025). Nanotechnology in oncology: Advances in biosynthesis, drug delivery, and theranostics. Discov. Oncol..

[76] Islam S., Ahmed M.M.S., Islam M.A., Hossain N., Chowdhury M.A. (2025). Advances in nanoparticles in targeted drug delivery–A review. Results Surf. Interfaces.

[77] Jia W., Wu Y., Xie Y., Yu M., Chen Y. (2025). Advanced Polymeric Nanoparticles for Cancer Immunotherapy: Materials Engineering, Immunotherapeutic Mechanism and Clinical Translation. Adv. Mater..

[78] Younas A., Wang S., Asad M., Al Mamun A., Majeed S., Sharif A., Zhou Q., Liu Y., Geng P., Shao C. (2026). Recent advances in cancer nanomedicine: From smart targeting to personalized therapeutics-pioneering a new era in precision oncology. Mater. Today Bio.

[79] Naik G.A.R.R., Gupta A., Datta D., More M., Roy A.A., Kudarha R., Hedayat P., Moorkoth S., Mutalik S., Dhas N. (2025). Synergistic combinational photothermal therapy-based approaches for cancer treatment. FlatChem.

